# Characterization of a newly isolated *Dhillonvirus* bacteriophage vB_EcoS-UDS3lw against *Escherichia coli* O157:H7

**DOI:** 10.1186/s13104-026-07856-w

**Published:** 2026-05-16

**Authors:** Yen-Te Liao, Abigail R. Arellano, Yujie Zhang, Vivian C.H. Wu

**Affiliations:** https://ror.org/03x7fn667 grid.507310.0 U.S. Department of Agriculture, Agricultural Research Service, Western Regional Research Center, Produce Safety and Microbiology Research Unit, Albany, CA 94710 USA

**Keywords:** *Dhillonvirus* phage, *E. coli* O157:H7, Depolymerase activity

## Abstract

**Objective:**

The aim of this study is to characterize a new *Dhillonvirus* bacteriophage that is lytic against *E. coli* strains, particularly *E. coli* O157:H7. Methods used for characterization included bacteriophage isolation, purification, bacteriophage morphology, host range test, one-step growth curve, whole-genome sequencing and analysis, and preliminary determination of depolymerase activity.

**Results:**

The complete genome sequence of *Escherichia* phage vB EcoS UDS3lw consists of double-stranded DNA, with a genome length of 44,071 base pairs and GC content of 54.67%. UDS3lw did not contain bacterial virulence genes, such as *stx*, or lysogenic genes in the phage genome. The phylogenetic analysis at the nucleotide level revealed that UDS3lw shared a close evolutionary relationship with *Escherichia* phages SM S22 and SSL-2009a, belonging to the *Dhillonvirus* genus. UDS3lw has a narrow host spectrum against *E. coli* O157:H7 and some non-pathogenic *E. coli* strains. Despite a small burst size of 2.4 PFU/cell on *E. coli* O157:H7 (RM9995), UDS3lw encodes a tail fiber protein (ORF 31) with depolymerase activity against non-pathogenic *E. coli* and most *E. coli* O157 strains. The findings provide insights into the genetic and biological diversity of the *E. coli-*infecting phage UDS3lw, which exhibits depolymerase activity and holds promise for further development as an anti-biofilm agent.

## **Introduction**

*Escherichia coli* (*E*. *coli*) is a Gram‑negative, rod‑shaped, facultatively anaerobic bacterium that typically colonizes the distal intestine of humans and other warm‑blooded animals as harmless commensals [1], and more than 50% of *E. coli* isolates are resistant to at least one antibiotic [2]. In contrast, Shiga toxin-producing *Escherichia coli* (STEC), particularly *E. coli* O157:H7, is one of the primary foodborne pathogens causing life-threatening illnesses, such as hemolytic uremic syndrome (HUS), thrombocytopenia, and kidney failure [3]. STEC O157 is one of the microbiota in ruminants’ GI tracts that frequently sheds into the surrounding environments and cross-contaminates food products and drinking water [3]. The bacterial pathogen can cause 97,000 illnesses, 3,270 hospitalizations, and 30 deaths annually in the United States [4]. While chemical-based antimicrobial interventions, such as weak acids and chlorine compounds, are typically used to prevent the spread of foodborne pathogens within food and the surrounding environment, recurring outbreaks and concerns about antimicrobial resistance development highlight the need for novel intervention technologies in the food industry [5]. Moreover, biofilm formation makes it even more challenging to eliminate the pathogen using conventional methods [6].

Virulent bacteriophages (or phages), the bacterial viruses with obligatory lytic infection, are gaining renewed interest as promising antimicrobial agents to combat bacterial contamination, particularly those prone to developing resistance [7]. Virulent phages are more favorable than conventional interventions because they are highly host-specific, ubiquitous in the environment for new phage isolation and antimicrobial development, and offer an alternative to address antibiotic resistance [8, 9]. Therefore, an increasing number of studies focus on characterizing virulent phages or their associated proteins as alternative antimicrobial agents to improve the efficiency of traditional intervention technology currently used in the food industry [10, 11]. However, several application challenges need to be overcome, and options to prevent bacterial resistance to phage infection are desired [12]. Therefore, in this study, we aim to characterize a new virulent phage that exhibits lytic infection of* E. coli* O157:H7 for future application.

## Materials and methods

### Phage isolation and purification

*Escherichia* phage vB_EcoS-UDS3lw (or UDS3lw) was isolated and purified with *E. coli* O157:H7 (RM 9995) from a sewage sample, collected from a water treatment plant at the University of California, Davis, using the previous method [13]. Liquid propagation was performed to prepare phage lysate for downstream analysis, as previously described [7].

### Genomic analysis

After phage DNA extraction using a Norgen phage DNA extraction kit (Thorold, ON, Canada), the TruSeq Nano DNA library prep kit (Illumina, USA) was used to prepare a DNA library, which was subsequently sequenced using a MiSeq Reagent Kit v3 (600-cycle) on the MiSeq platform (Illumina, San Diego, CA, USA). Approximately 0.31 million paired-end (2 x 250 bp) sequence reads were generated and subjected to Trimmomatic with Q30, followed by genome assembly using SPAdes (v.3.15.3). Only one resulting contig was obtained and subjected to BLASTn to confirm the phage genome and PhageTerm analysis (Galaxy v.1.0.12) [14]. Later, the annotation was conducted using the Prokka pipeline (Galaxy 1.13) with default settings [15] and was confirmed against the Universal Protein Resource (UniProt) database [16]. The final phage sequence was screened for virulence and antibiotic resistance genes using the web servers VirulenceFinder v2.0 [17] and ResFinder 4.7.2 [18], respectively. The genome map was generated using the CGview server beta (https://proksee.ca/). The DeepPL tool was utilized to determine the lifecycle of UDS3lw [19]. The DePolymerase Predictor server (https://timskvortsov.github.io/WebDePP/) was used to predict ORF-encoding proteins as potential depolymerase enzymes. The final annotated sequence of UDS3lw was deposited in the National Center for Biotechnology Information (NCBI) database, with accession number PQ658967.1.

### Comparative genomics

Whole-genome phylogenetic analysis of phage UDS3lw with closely related reference phages was conducted using the Virus Classification and Tree Building Online Resource (VICTOR) webserver at the nucleic acid (D0) level [20]. JSpeciesWS was used to perform a pairwise genome comparison by calculating average nucleotide identity (ANIb) between UDS3lw and closely related reference phages [21]. Maximum-likelihood phylogenetic analysis was conducted on amino acid sequences of tail proteins, such as tail fiber and tail spike, with 500 bootstrap replicates [22].

### Transmission electron microscopy (TEM)

To observe phage morphology, CsCl-purified phage UDS3lw was subjected to negative staining and examined under a transmitted electron microscope (TEM, FEI Tecnai G2), as previously described [13].

### One-step growth curve

A one-step growth curve was performed on *E. coli* O157:H7 (RM 9995) using the previously described method [13]. In brief, 0.2 mL of an overnight culture was subcultured into 19.8 mL Lof tryptic soy broth (TSB; Becton, Dickinson, Sparks, MD) and incubated for 2 h at 37℃ before the adding of 0.2 mL of 10 mM CaCl_2_ and phage UDS3lw at a multiplicity of infection (MOI) of 0.01. After a 10-min incubation, the mixture was centrifuged at 8,000 ⋅xg for 5 min at 4℃, and the pellet was washed twice before resuspension in 20 mL of TSB. Finally, 0.3 mL of the resuspended culture was added to 29.7 mL of TSB, and 1 mL of the mixture was obtained without filtration to determine the phage-infected bacterial cells using a double-layer plaque assay, while the remaining was incubated at 37℃ with shaking for 70 min; a 1 mL sample was taken and filtered every 10 min. Each sample was subjected to a double-layer plaque assay with fresh overnight culture to estimate the latent period and burst size of phage UDS3lw. The experiment was conducted in 3 replications.

### Host range test and efficiency of plating (EOP)

The host range of UDS3lw was conducted against various STEC, non-pathogenic *E. coli*, and *Salmonella* strains using a spot test assay, as previously described [13]. The susceptible bacterial strains were then used to determine the infectivity of UDS3lw using the efficacy of the plating assay, as previously described [23]. Briefly, double-layer plaque assays were carried out against each host and the resulting plaques were enumerated to determine the phage titer. EOP was calculated by the ratio of phage titer on a test bacterium versus the primary bacterial host. High production efficiency is EOP ≥ 0.5, medium production efficiency is 0.5 > EOP ≥ 0.1, low production efficiency is 0.1 > EOP > 0.001, and the inefficiency of phage production is EOP ≤ 0.001. The experiment was conducted in 3 replications.

### In vitro antimicrobial activity in lysogeny broth

A bacterial culture of *E. coli* O157:H7 RM9995 was prepared in 10 mL TSB at 37 °C for 20 h, then diluted in lysogeny broth (LB; Invitrogen, Carlsbad, CA, USA) to approximately 3.8 log CFU/mL (final concentration), and 20 mL of this was dispensed in sterile 50-mL conical tubes. Meanwhile, phage UDS3lw was diluted in SM buffer and then added accordingly to each tube at MOIs of 35, 350, and 3,500. For the control group, an SM buffer equal in volume to the phage solution was used. All the tubes were incubated at 25 °C for 6 h, followed by bacterial plating on Sorbitol MacConkey agar (SMAC; BD, Franklin Lakes, NJ, USA) overlayered with thin tryptic soy agar. The experiment was conducted in 3 replications.

## Results and discussion

### Genomic characteristics

**Fig. 1:**
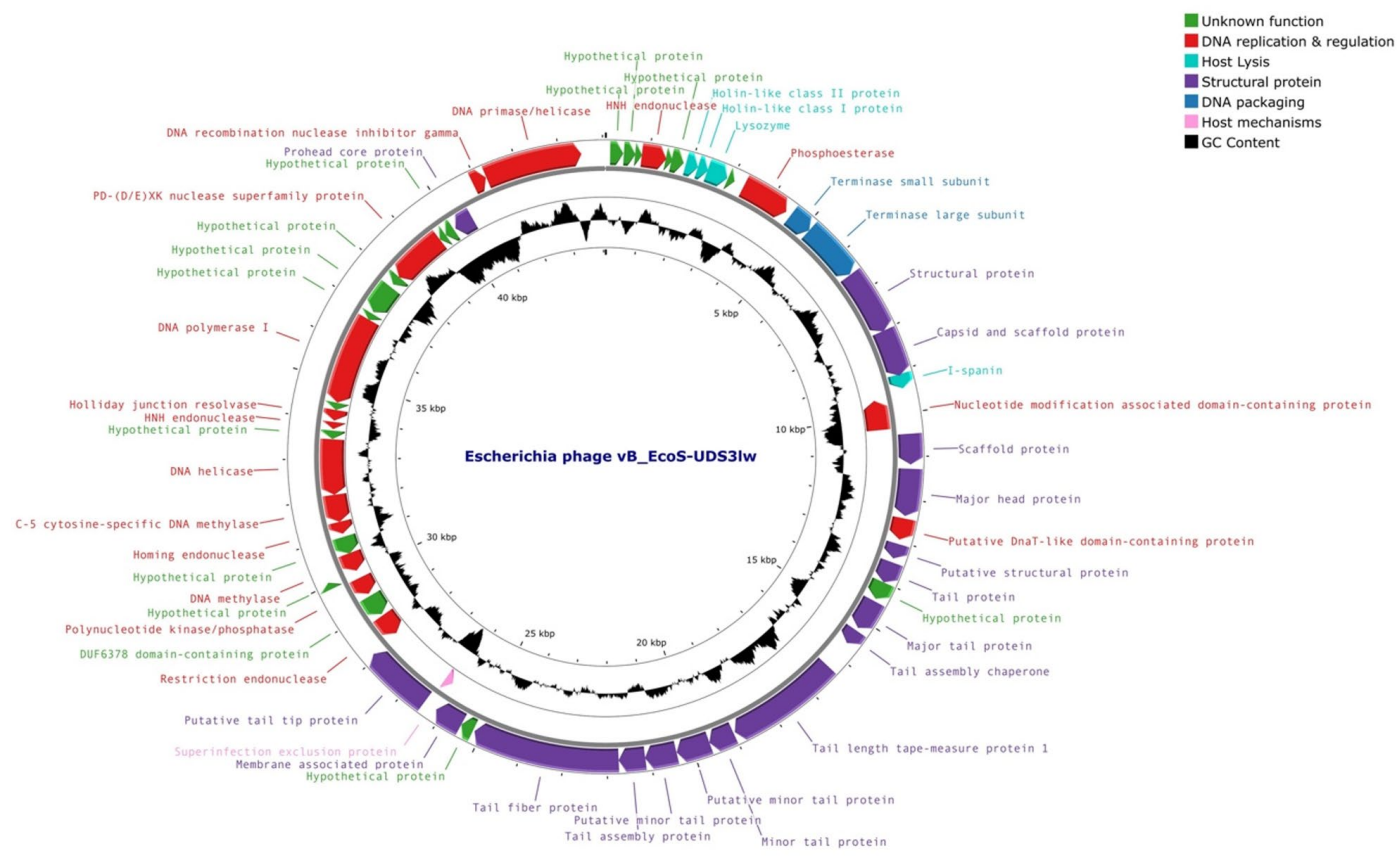
The genome map of *Escherichia* phage vB_EcoS- UDS3lw. The annotated ORFs are colored red (DNA replication and regulation), turquoise (host lysis), purple (structural proteins), blue (DNA packaging), pink (host mechanisms), and green (unknown function) on the map. The center of the genome map provides % GC content (black)

Phage UDS3lw had a double-stranded DNA genome, with a size of 44,071 bp and an average GC content of 54.67%. The results showed that phage UDS3lw had 58 open reading frames (ORFs), 40 of which were annotated with predicted functions, such as DNA replication and regulation (*n* = 16), host lysis (*n* = 4), host mechanisms (*n* = 1), DNA packaging (*n* = 2), and structural proteins (*n* = 17) (Fig. 1). The phage genome did not harbor antimicrobial resistance or virulence genes. Meanwhile, PhageTerm analysis indicated that UDS3lw employs headful DNA packaging with a preferred packaging (*pac*) site [24]. DeepPL analysis further confirmed that UDS3lw is a virulent phage with a strictly lytic lifecycle.

**Fig. 2:**
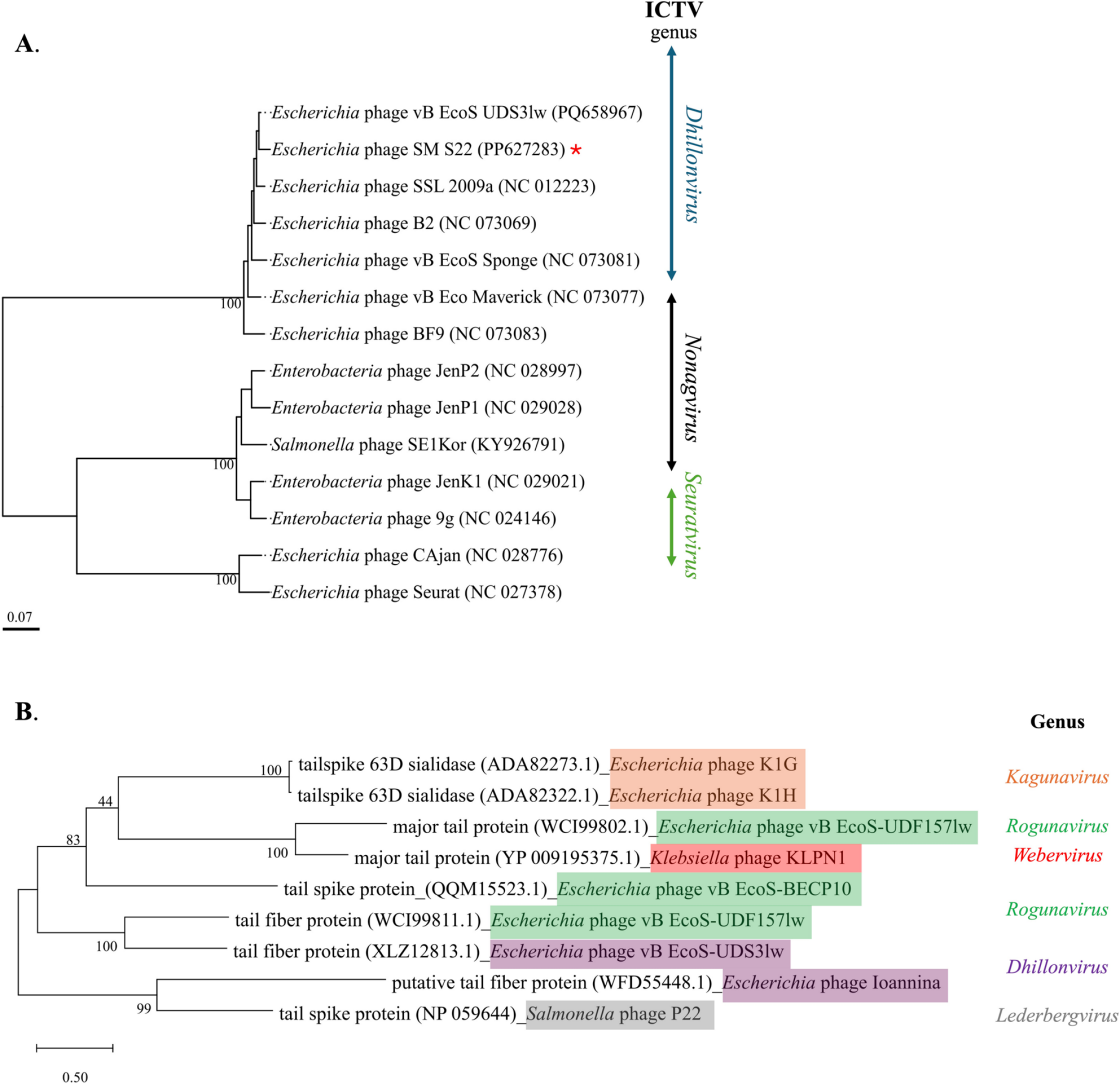
Phylogenetic analysis. **A** Phylogenetic tree of the whole-genome sequence of UDS3lw and closely related reference phages belonging to the *Dhillonvirus* genus at the nucleotide level using VICTOR (formula d0), and **B** Maximum-likelihood phylogenetic analysis of tail proteins at the amino acid level, including tail fiber and tail spike, associated with depolymerase activity across diverse phage genera. Red asterisk indicates the phage belongs to the *Dhillonvirus* genus by the NCBI classification, but has not been confirmed by the ICTV. The accession numbers for reference phages and various tail proteins are provided in parentheses

VICTOR analysis showed that UDS3lw was in the same cluster as the reference phages classified in the *Dhillonvirus* genus by the International Committee on Taxonomy of Viruses (ICTV) (Fig. 2A). Moreover, the phage showed high genomic similarity to *Dhillonvirus*-like *Escherichia* phage SM S22, classified by NCBI but not ICTV yet, and *Escherichia* phage SSL-2009a, classified by the ICTV, with an ANIb of 92.6% (91.35% query coverage) and ANIb of 90.11% (89.70% query coverage), respectively. This finding likely suggests that UDS3lw is a new member of phages belonging to the *Dhillonvirus* genus [25]. The *Dhillonvirus* phages typically exhibit a relatively narrow host range, particularly against *E. coli* strains, but rarely against *Salmonella* strains [26, 27]. For example, *Escherichia* phage Ioannina infected multiple *E. coli* isolates of clinical or environmental origins but not *E. coli* O157:H7 [27]. In contrast, UDS3lw primarily targeted *E. coli* O157:H7 and some non-pathogenic *E. coli* strains. Phylogenetic analysis of the tail proteins, which mediate host recognition and binding, showed that UDS3lw encoded a tail fiber protein with high amino acid sequence similarity to that of the *Rogunavirus* phage UDF157lw (Fig. 2B). The results are consistent with the similarly narrow host ranges between the two genomically diverse phages [13]. Additionally, the tail fiber protein of *Rogunavirus* phages, such as EcoS_BECP10, use O-antigen on lipopolysaccharide (LPS) for bacterial binding [28, 29]. On the other hand, the *Dhillonvirus* phage DH23 utilizes O-antigens on *E. coli* membrane to initiate phage infection [26]. Collectively, these findings support the hypothesis that UDS3lw similarly engages O-antigens on the susceptible *E. coli* strains, including STEC O157. In addition to their role as receptor-binding proteins that mediate host recognition and adsorption, these tail proteins also exhibit depolymerase activity, enabling the degradation of bacterial polysaccharides [29].

### Biological characteristics

**Fig. 3:**
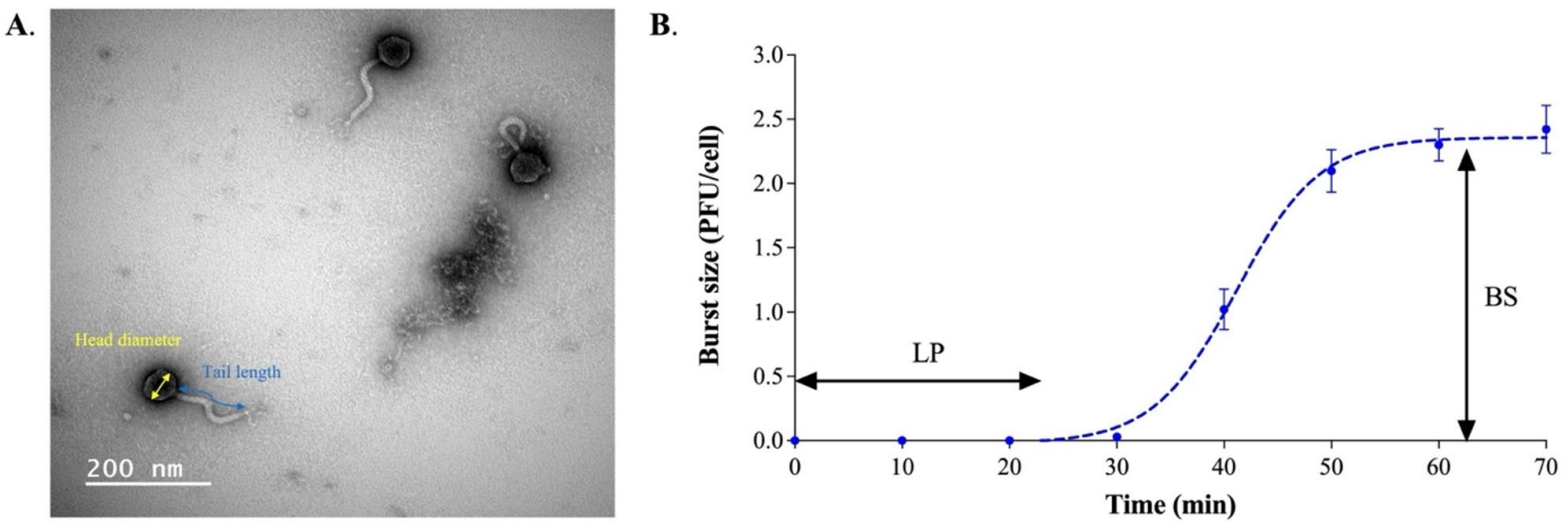
Biological characteristics of phage UDS3lw. **A** Transmission electron microscopy image of phage UDS3lw contained a capsid with 53 ± 1 nm in diameter and a long non-contractile tail of 142 ± 5 nm in length. **B**A one-step growth curve for *E. coli* O157:H7 RM 9995 showed a latent period (LP) of 20 min and a burst size (BS) of 2.5 PFU/cell

Regarding biological characterization, transmission electron microscopy showed that phage UDS3lw had an icosahedral head of 53 ± 1 nm in diameter and a long non-contractile curled tail of approximately 142 ± 5 nm in length (Fig. 3A). Additionally, the one-step growth curve results demonstrated that UDS3lw had a 22-min latent period and an average burst size of 2.4 PFU per infected cell against *E. coli* O157:H7 RM9995 (Fig. 3B).

Regarding host range, phage UDS3lw showed lytic activity against non-pathogenic *E. coli* and STEC O157, but no infection on non-O157 STEC, other pathogenic *E. coli*, or *Salmonella* strains (Table 1). Moreover, UDS3lw exhibited a range of productive infection efficiencies, from high to low, across different *E. coli* O157:H7 strains, while showing high productive infection against non-pathogenic *E. coli*, including ATCC13706 and ATCC8739. UDS3lw may produce a larger burst size when infecting non-pathogenic *E. coli* strains with high EOP, such as ATCC13706 and ATCC8739, resulting in higher phage titers [30].

**Fig. 4:**
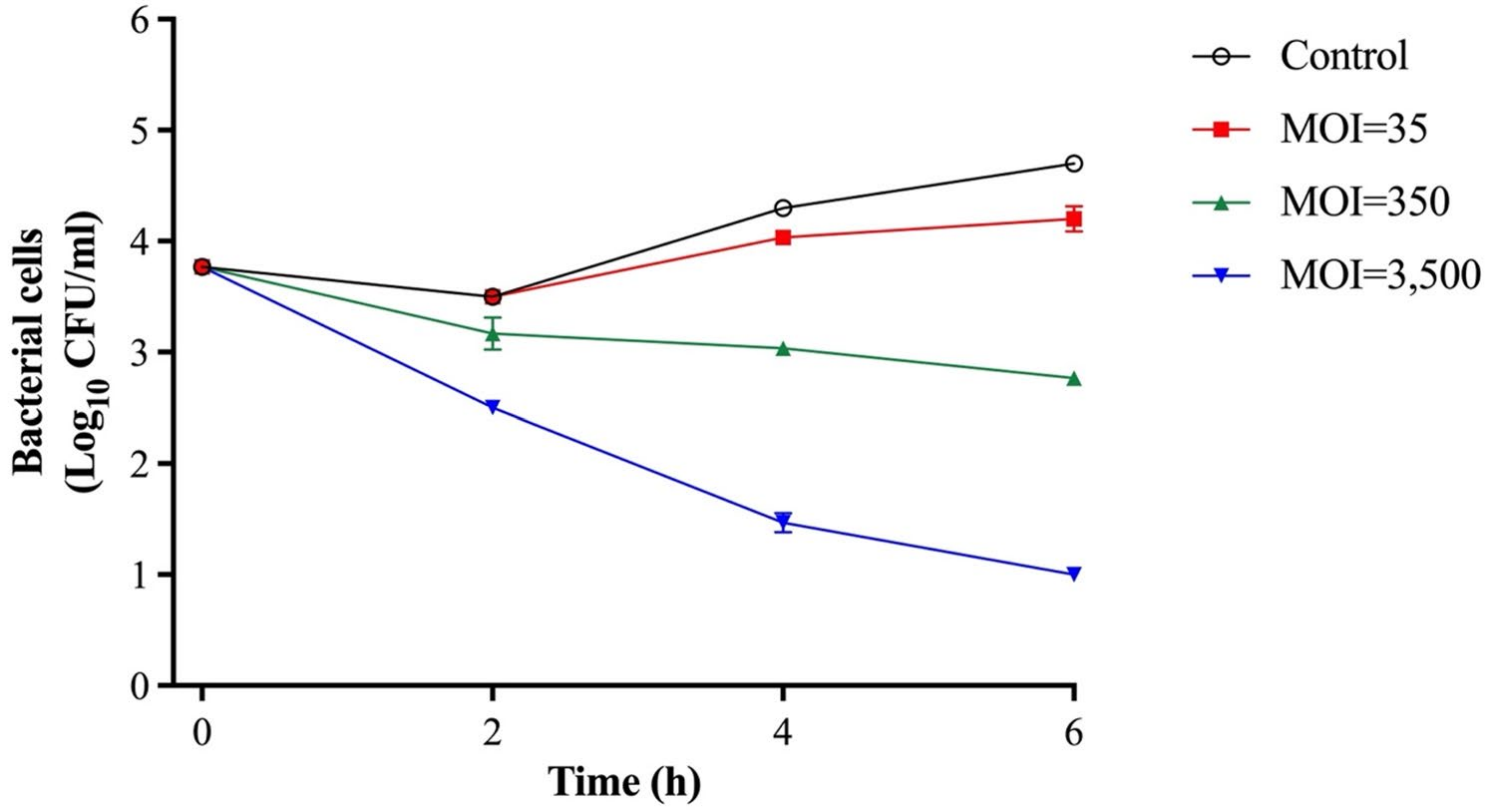
The antimicrobial activity of UDS3lw against *E. coli* O157:H7 (RM9995). Different MOIs of the phage (35, 350, and 3,500) were used for the treatment in lysogeny broth at 25 °C for 6 h. The error bars show the standard error of the mean (SEM)

In the antimicrobial test, UDS3lw, with a low MOI of 35, did not reduce *E. coli* O157:H7 but did suppress bacterial growth over the treatment period (Fig. 4). This may have resulted from the phage’s small burst size. However, the bacterial reductions were increased when higher MOIs of 350 and 3,500 were used, causing 1.9 log and 3.7 log reduction, respectively, compared to the control group at the 6-h time point (Fig. 4). The pronounced bacterial reduction at these high MOIs may also have been caused by lysis from without [31]. Although UDS3lw alone may not be sufficient to reduce pathogenic *E. coli* O157:H7 at low concentrations, its antimicrobial efficacy could be enhanced by combining multiple phages [32]. Furthermore, the low biological capability of phage DCS3lw to produce phage progeny may reflect a trade-off in its evolutionary history for persistence in the environment [33].

**Table 1:** Host range, efficiency of plating (EOP), and depolymerase activity against various bacterial strains

Strains	Strain ID	stx	EOP*	Depolymerase activity
Non-O157 STEC	O26, O45, O103, O111, O121, and O145	–	R	–
STEC O157	*E. coli* O157:H7 (RM18959)	*stx1&2*	0.04	No
	*E. coli* O157:H7 (ATCC35150)	*stx1&2*	0.11	Yes
	*E. coli* O157:H7 (ATCC43888)	No	< 0.001	No
	*E. coli* O157:H7 (RM18419)	*stx2*	< 0.001	Yes
	*E. coli* O157:NM (RM11781)	*stx2*	< 0.001	Yes
	*E. coli* O157:H7 (RM19259)	*stx2*	0.62	Yes
	*E. coli* O157:H7 (RM9995)	*stx2*	H	Yes
Non-pathogenic *E. coli*	ATCC13706	No	2.16	Yes
	TVS353	No	R	-
	ATCC8739	No	2.66	Yes
Enterotoxigenic *E. coli*	O117 (RM6844), O25 (RM6850), O159 (RM6856), O19 (RM6841), O119 (RM2169), & O29 (RM2172)	–	R	–
Enteropathogenic *E. coli*	O119 (RM2169)	–	R	–
Enteroinvasive *E. coli*	O29 (RM2172)	–	R	–
*Salmonella*	*Salmonella* Enteritidis (PT30)	–	R	–
	*Salmonella* Typhimurium (14028)	–	R	–
	*Salmonella* Infantis (BAA-1675)	–	R	–

* EOP was calculated by the ratio of phage titer on a test bacterium versus the primary bacterial host. High production efficiency is EOP ≥ 0.5, medium production efficiency is 0.5 > EOP ≥ 0.1, low production efficiency is 0.1 > EOP > 0.001, and the inefficiency of phage production is EOP ≤ 0.001. H means the bacterial host used for the phage isolation and propagation. R means the bacterial strain resistant to the phage infection without any bacterial lysis.

**Fig. 5:**
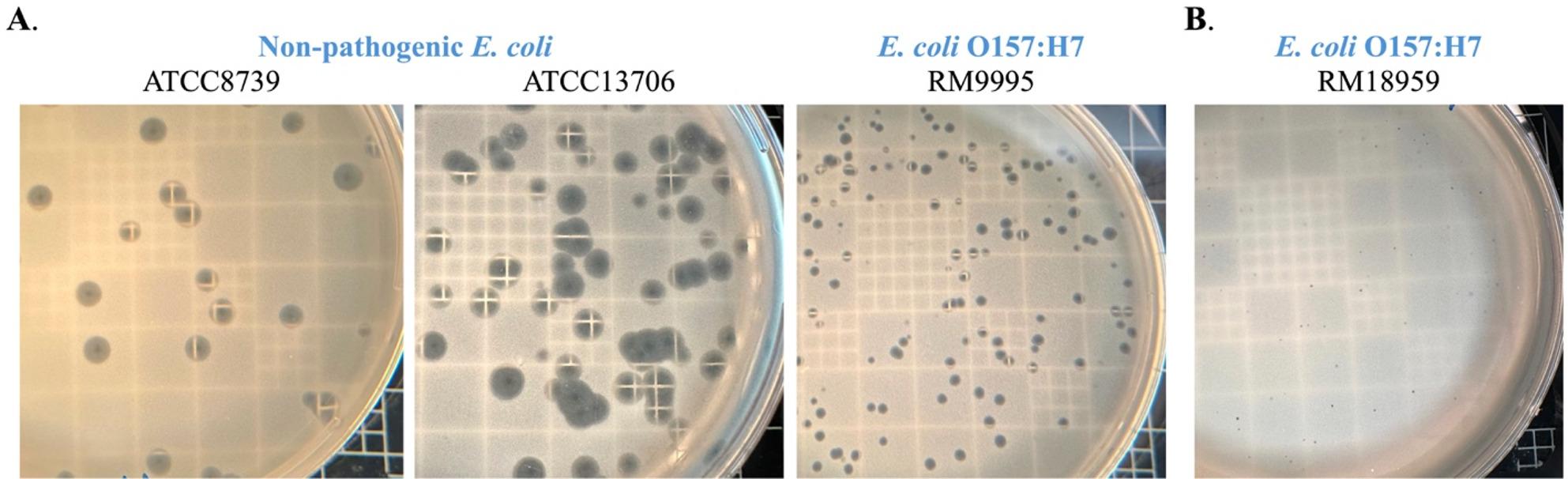
Plaque morphologies of phage UDS3lw. **A** Large and halo-surrounding plaques were produced against non-pathogenic *E. coli* ATCC8739, ATCC13706, and *E. coliO157:H7 (RM9995)*, and **B** small plaque sizes were produced against *E. coli* O157:H7 (RM18959) on plaque-assay plates with top soft agar

Interestingly, UDS3lw produced different sizes of large plaques, with a clear center surrounded by a halo zone, upon infection against most *E. coli* O157:H7 and non-pathogenic *E. coli* strains (Fig. 5A). However, the phage did not produce similar large plaques against *E. coli* O157:H7 (RM18959 and ATCC43888) (Fig. 4B; Table 1). The large, halo-surrounding plaques showed signs of depolymerase activity (Fig. 5A); the enzyme was primarily produced during the phage infection by cleaving exopolysaccharides and lipopolysaccharides on the bacterial host membrane through its tail fibers or tail spikes, and it was also used to disperse biofilm structure [28, 29]. A previous study found that long-tail phages, such as those belonging to the *Dhillonvirus*, *Nonagvirus*, or *Seuratvirus* genus, use the O-antigen as their primary receptor, which allows them to access the secondary receptor and initiate infection [34]. Based on the results of the DePolymerase Predictor in this study, the tail fiber protein (ORF_31) of phage UDS3lw was predicted to be a depolymerase and showed a high evolutionary relationship with the counterfeit in phage UDF157lw (Fig. 2B). In our previous study, phage UDF157lw reduced 24-h and 48-h *E. coli* O157:H7 biofilm in a 24-well plate model [13]. Unlike UDF157lw, phage UDS3lw showed depolymerase activity against non-apthogenic *E. coli* ATCC13706 but not *E. coli* O157:H7 RM18959. These findings suggest that phage UDS3lw may not use the same O-antigens as UDF157lw for bacterial binding and depolymerase activity during infection. Further investigation is required to elucidate alternative binding mechanisms involving different phage receptor-binding proteins and cell-membrane polysaccharides.

## Conclusion

To conclude, phage UDS3lw is a new member of *the Dhillonviruses* phages, with a host range against several non-pathogenic *E. coli* strains and various *E. coli* O157:H7 strains. The phage genome does not contain bacterial virulence, lysogenic, or antibiotic-resistance genes. Despite its small burst size, UDS3lw may be useful for phage-bacterial interaction studies by examining the bacterial transcriptomic response to a virulent phage treatment with a comparatively weak lytic effect. Most of all, UDS3lw encodes a tail fiber protein with depolymerase activity against most selected *E. coli* strains, including *E. coli* O157:H7, in this study. The findings provide insights into the genetic diversity of an *E. coli*-infecting phage, supporting the future development of depolymerase as an alternative antimicrobial agent.

### Limitation

The current study did not provide stability tests, because those results are more meaningful for application in future studies.

## Data Availability

The genome sequence of Escherichia phage vB_EcoS-UDS3lw was deposited at the NCBI database under GenBank accession number # PQ658967.1.

## Abbreviations

STEC: Shiga-toxin producing *E**. coli*
HUSH: Hemolytic uremic syndrome
ORF: Open reading frame
NCBI: National Center for Biotechnology Information
ANIb: Average nucleotide identity
TEM: Transmission electron microscopy
TSB: Tryptic soy broth
MOI: Multiplicity of infection
EOP: Efficiency of plating
ICTV: International Committee on Taxonomy of Viruses
LP: Latent period
BS: Burst size
VICTOR: Virus classification and tree building online resource
